# Effect of storage conditions on lignocellulose biofuels properties

**DOI:** 10.1038/s41598-024-66118-6

**Published:** 2024-07-02

**Authors:** Małgorzata Wzorek, Anna Król, Robert Junga, Joanna Małecka, Ersel Yilmaz, Alicja Kolasa-Więcek

**Affiliations:** 1 Department of Environmental and Process Engineering, Opole University of Technology, Mikołajczyka Str. 5, 45-271 Opole, Poland; 2 Department of Thermal Engineering and Industrial Facilities, Opole University of Technology, Mikołajczyka Str. 5, 45-271 Opole, Poland; 3 Department of Mechanics and Machine Design, Opole University of Technology, Mikołajczyka Str. 5, 45-271 Opole, Poland; 4 Department of Biosystems Engineering, Aydin Adnan Menderes University, South Campus, 09020 Aydin, Turkey; 5 Institute of Environmental Engineering and Biotechnology, University of Opole, Dmowskiego Str. 7, 45-365 Opole, Poland

**Keywords:** Agro-biomass, Pellets, Storage, Artificial atmospheric conditions, Mechanical properties, Environmental impact, Environmental monitoring, Atmospheric chemistry

## Abstract

This article examines the effects of different storage conditions on selected physicochemical properties of three types of agro-biomass pellets: sunflower husks, wheat straw and hemp hurds, and wood pellets. The tests were carried out in a climatic chamber, which allows simulation of real storage conditions, i.e. conditions with high air humidity and variable (±) ambient air temperatures. The results showed higher degradability of agro-biomass pellets compared to woody biomass. The pellets degraded to a less extent at varying  ± temperatures than at high humidity (90% RH). After complete moisture saturation, durability decreases for agro-pellets by an average of 9%, while after freezing and defreezing for sunflower husk pellets and woody pellets durability decreases by 2%, and for hemp hurd pellets by 11%. In contrast, strength-by-dropping index for agro-pellets decreased by 20% after being in the environment (30 °C and 90%RH) and 15% under varying temperature conditions. No change in the energy parameters of all pellets in the dry matter was noted. On the other hand, an increase in the moisture content of pellets when they are stored under different environmental conditions results in a decrease in calorific value.

## Introduction

Increasing energy demand and environmental considerations have led to the increasing use of renewable energy sources, including biomass. Biomass can be used in power plants and combined heat and power plants, but also in domestic furnaces in the form of chunks, chips, or processed into pellets, which is gaining more interest.

The global demand for pellets in the EU, Canada, and the U.S. is estimated to increase to about 26 million metric tonnes in 2027, compared to an estimated 18.2 million metric tonnes consumed in 2020^1^. However, the industrial trade for wood pellets involves the international bulk transport of more than 10 million tonnes annually^2^.

This poses a major challenge for the power generation industry in terms of providing sufficient fuel to sustain the combustion process and storing such large volumes of fuel. Biofuels not only need to possess suitable energy properties but also physical properties that facilitate their transportation, storage, and easy dispensing into the combustion chamber. Consequently, pelletizing and briquetting techniques are utilized to densify, homogenize, and form solid biomass^3,4^.

The processes mentioned above enable an increase in the specific density of biofuels, which is crucial for reducing their transportation and storage costs. Simultaneously, these processes result in a significant enhancement in energy density compared to unprocessed biomass. Compaction processes also allow the utilization of biomass feedstocks in biofuel production, especially those whose original form makes it impossible to use directly in energy processes. Through molding methods, various biomass materials and other additives, such as binders, can be utilized to produce biofuels meeting the quality parameters required by current standards and, more importantly, expected by end users^5,6^.

The physical properties of biomass pellets and, above all, the mechanical parameters depend on the type of biomass, largely on the internal structure, the binding mechanisms and friction of the material in the pellet channel, the porosity, the size and arrangement of the grains and the parameters of the compaction process such as temperature, pressure and moisture of materials^4^.

The storage of biofuels in inappropriate conditions can negatively affect their quality, and the diversity of biomass wastes makes them differently susceptible to various destructive factors, which can be, for example, climatic factors, i.e., precipitation, varying temperatures, freeze–thaw cycles and the length of time the fuels are exposed to certain weather conditions.

Therefore, it is important to consider the effects of storage and handling on the physical properties of the biomass pellets. A few publications have investigated the effects of long-term storage on the mechanical and chemical properties of wood pellets^7–9^. It has been proven that the mechanical integrity of pellets can be compromised during storage or transport. Lehtikangas^9^ studied pellets made out of sawdust, logging residues and bark during the 5 months storage in plastic bags in an unheated barn. Apart from a negative effect on durability, microbial growth was also observed. A similar effect was detected by Kymäläinen et al.^5^ for torrefied wood, charcoal, and thermally treated pellets that were stored in both covered and uncovered areas. Tests indicate that 99% of uncovered samples showed visible fungal growth, but only 20% of the covered samples did. Additionally results demonstrated that storage had varying effects on the durability and moisture absorption of the pellets. Steam exploded pellets were found to have more favorable properties than torrefied and untreated pellets.

It was found that the pellets weaken over time and can generate significant amounts of dust, which, in turn, raises the likelihood of fires and explosions^10^.

Compared to woody biomass, there has been less extensive reporting on the storage of agro-waste biomass. Due to increasing interest in utilizing more varied biomass types, attention should also be paid to the parameter stability of pellets made from agricultural waste. This is due to the fact that woody biomass has a different structure compared to, for example, straw, fibrous residue, stalks, or husks. For example, woody biomass generally contains on average (typically) about 25% lignin, the content of which can still increase during storage due to the decay of the readily available carbohydrate fraction^2^. On the other hand, agricultural wastes contain much less of it, for example, cereal straws and corn stalks < 20%, rice straw about 10%, which has an impact on the lower strength parameters of the pellets made from them.

Due to the lack of information available regarding the impact of different storage conditions on the physical properties of agricultural waste pellets, a study was conducted to assess the effects of various atmospheric factors, such as increased relative humidity and varying ambient temperatures, on pellet quality. The study involved four selected types of pellets and was conducted under laboratory conditions, where present atmospheric conditions were simulated in an environmental chamber. Simulating artificial conditions allowed for a more rapid assessment of environmental effects compared to tests conducted in natural storage environments.

## Materials and methods

### Materials

Three types of pellets made from different types of agro-biomass, i.e. sunflower husks, wheat straw and hemp hurds, were tested.

A 100 kg/h pressure pellet mill equipped with two movable rollers and a variable flat die with Ø6 and Ø8 mm holes was used for manufacturing. Due to the type of material, a die with Ø8 mm holes was used to produce sunflower husk pellets, and Ø6 mm for other materials.

The grinding and moisture content used for palletizing depended on the type of material and its susceptibility to grinding, i.e., the grinding of sunflower husk was ≤ 60 mm, wheat straw ≤ 50 mm, and hemp hurds ≤ 2 mm. The initial moisture content of the materials ranged from 8 to 12%, and additionally, the materials were moistened during palletizing to achieve better compaction of the produced pellets.

Industrial pine wood pellets were also examined for comparison.

The pellets have been given appropriate designations:

- Pellet A—pellets made of sunflower husks.
- Pellet B—pellets made of wheat straw.
- Pellet C—pellets made of hemp hurds.
- Pellet D—pellets made of pine sawdust.

After manufacture, the pellets were seasoned for a period of 7 days and then tested.

### Methods

#### Simulation of pellet storage in different environmental conditions

**Artificial climatic conditions.** The study of the effects of different environmental conditions on pellet quality was carried out in a climatic/environmental chamber, which allows simulating actual storage conditions by cycling i.e., short- and long-term effects of temperature and air humidity on pellets, and even evaluating their behavior in exacerbated conditions compared to real conditions.

Given the climatic conditions in central Europe, it was decided to simulate spring and autumn conditions with high moisture content in the air and winter conditions with variable (±) ambient air temperatures.

A Weiss ClimeEvent climate chamber was used in the study, which allows samples to be tested under temperatures ranging from −70 to + 180 °C and relative humidity ranging from 10 to 98%.

All types of pellets in quantities of 2 kg were placed in a chamber and subjected to environmental tests.

The study was conducted in two measurement cycles:

1. Evaluating the impact of the moisture adsorption capacity of the pellets—humidity test;

The pellets were kept in a chamber at a temperature of 30 °C and a relative humidity of 90% until saturation was reached, that is, a constant value of the weight of the samples was obtained. The change in the weight of the samples was measured in 1-h cycles. The change in the increased weight of the sample was expressed as an increase in the moisture content of the pellets. The test was conducted for 48 h and repeated twice.

(2) Evaluating the impact of temperature changes—freezing–thawing cycles—simulation of winter conditions.

Pellets were then subjected to freezing–thawing cycles (40 times). That number of cycles was to simulate the average time with ambient temperatures below 0 °C in winter. One cycle involved freezing a sample down to − 10 °C which was followed by thawing and bringing the temperature to + 10 °C. Each cycle lasted 4.5 h, and freezing and thawing were carried out as follows: transitioning from 10 to − 10 °C took 15 min, staying at − 10 °C lasted for 120 min, then transitioning to 10 °C took 15 min, and staying at 30 °C lasted for 120 min. The test underwent two repetitions.

**Leachability of harmful components**. The pellets after the moisture and temperature test were evaluated for the potential leachability of harmful components from them, i.e. heavy metals to assess the potential impact of environmental factors occurring during pellet storage.

Heavy metal leaching was performed in accordance with EN 12457-4:2006. Samples of pellets (100 g) were shaken with water during 24 h at the liquid-to-solid (L/S) ratio equal to 10 (weight). Leachates were prepared at 20 ± 5 °C. When the shaking was finished, the containers were left to stand for about 15 min to let the solid particles settle down. The extracts were then filtered: the filter pore diameter was 0.45 μm. The leachates were then subjected to tests to find the content of the pollutants^11^. The leaching test was repeated twice.

The pH of the aqueous extracts was measured by potentiometric titration and heavy metal content was determined in accordance with EN ISO 11885:2009 using an ICP-OES spectrometer (5800 Agilent).

#### Mechanical properties

Mechanical properties of the pellets were carried out for fresh pellets and after environmental tests, i.e. moisture test and temperature test. Parameters such as durability and strength-by-dropping were studied.

**Durability**. A measure of the resistance of compacted fuel to shock and/or abrasion following handling and transportation processes is mechanical durability.

The tests were conducted in accordance with EN ISO 17831-1:2015, according to which a 500 g ± 10 g pellet sample is placed in a tester chamber of standardized dimensions and subjected to centrifugation at 50 ± 2 rpm for 10 min. After stopping the tester, the sample is sieved on a sieve with 3.15 mm hole diameter. The material remaining on the sieve is weighed, and the durability Du is calculated according to the equation:

$${D}_{U}=\frac{{m}_{A}}{{m}_{E}} \cdot 100 \%$$

where m_A_- mass of sample before test, g. m_E_ – mass of samples after test, g

The mechanical durability of was determined using five repetitions for each type of pellet.

**Drop strength**. In the strength-by-dropping test, sample is dropped down twice from 1.5 m against a concrete surface. The strength-by-dropping factor is calculated from the formula^12^:

$${W}_{Z}=\frac{\text{N}}{{N}_{1}} \cdot 100 \%$$

where N – number of pellets, which were subjected to testing, pcs. N_1_ – number of pellets, which survived with no damage, pcs. The test was conducted for all pellets in five repetitions.

#### Energy properties

The energy parameters of the pellets were analyzed using methods described in Table 1. The Elementar Vario MACRO Cube was used to determine the analysis of C, H, N and S in the pellets. The higher heating value (HHV) was measured using the IKA C5000 bomb calorimeter. The research repetitions adhered to the normative requirements for each analysis.

**Table 1:** Analysis of energy parameters.

Moisture, M	ISO 18134
Volatile matter, VM	EN ISO 18123
Fixed carbon, FC	calculated by difference
Ash, A	EN ISO 18122
Elementary analysis: C, H, N, S, O	EN ISO 16948, EN ISO 16994 (S); calculated by difference (O)
Higher Heating Value, HHV	EN ISO 18125, ISO 1928

#### Scanning electron microscopy (SEM) images of pellets

Images of the pellets before and after environmental testing were taken by scanning electron microscopy (SEM) using a TESCAN VEGA 4 microscope equipped with SE (secondary electron), BSE (backscattered electron) detectors and an (EDS) energy dispersive X-ray microanalysis system.

In order to obtain adequate electrical conductivity, the surface of each sample was sprayed with a thin layer of gold before SEM imaging. The SEM images were operated with the same parameters—magnification of about 30–50x.

## Results and discussion

### Simulation of pellet storage in different environmental conditions

The moisture content of biomass materials does not only affect the pelletization process itself, and thus the proper compaction of the material but also the mechanical strength of the pellets immediately after the process. In the case of storage, the ability to absorb moisture from the environment is important because pellets may be exposed to rain or high air humidity during transportation and storage.

In order to evaluate the effects of atmospheric factors such as air humidity and temperature on agro-biomass pellets, they were subjected to environmental tests in artificial environmental conditions.

Since Deng et al.^13^ found that humidity levels between 20 and 60% did not cause significant degradation for woody pellets, the study was conducted at higher RH values of 90%.

**Figure 1:**
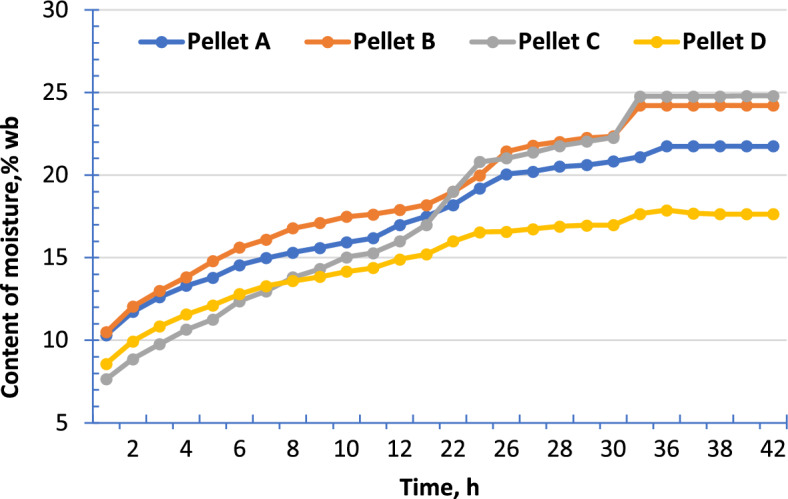
Change in pellet moisture content over time during storage at 30 °C and 90% RH.

The moisture content of the pellets as a function of exposure time in a controlled environment is shown in Fig. 1.

Simulation tests of storage at 30 °C and 90% RH humidity showed that the pellets tested, on average, absorb less than 25% moisture under such conditions.

The ability of pellets to absorb moisture from the environment is mainly dependent on the type of material and its hygroscopic properties. Moisture content increased by over 21.75% for Pellet A, 24.21% for Pellet B, and up to 24.88% for Pellet C within a period of 34 h. For example, Yu et al.^14^ obtained equilibrium moisture content for wheat straw above 24%, and for canola straw pellets 34.15%.

The lowest moisture content values were obtained for woody biomass pellets (Pellet D)—17.64%. The lower absorption of water in a high humidity environment by woody pellets compared to agro-pellets is also confirmed by other studies^14,15^.

In order to meet the standards for solid biofuels, it is necessary for the moisture content of pellets to be below 10% (classified as A1) and for lower quality classes, the upper limit for moisture content should not exceed 15%. Stored pellets after the temperature test did not exceed the value of 15%, unlike in the moisture test.

**Figure 2:**
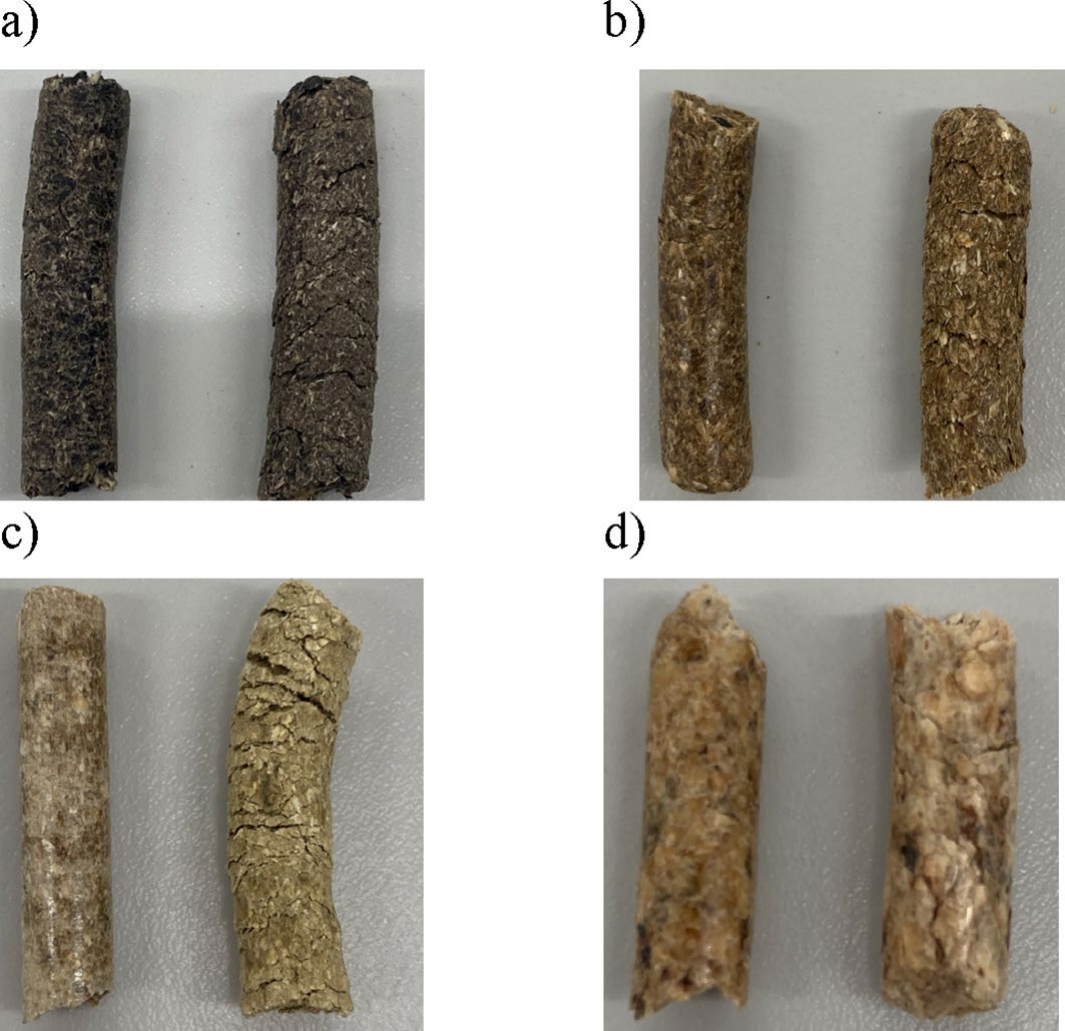
Pellets before (left side) and after artificial degradation in climatic chamber (right side: (**a**) Pellet A; (**b**) Pellet B; (**c**) Pellet C; (**d**) Pellet D.

Figure 2 shows the changes in the appearance of the pellets before and after the test in a climatic chamber simulating the effects of temperature and humidity on the pellets.

Studies have shown that exposure of agro-pellets to varying weather conditions causes them to swell and delaminate. After the moisture test, the pellets increased their diameter by an average of 2 mm. For the 20 pellets of each type tested after this test, the average diameter for Pellet A was 10.28 mm (8.5 mm diameter before test); Pellet B 6.85 mm (6.85 mm before test); Pellet C 8.02 mm (6.02 mm diameter before test) and Pellet D 6.33 mm (9.05 mm diameter before test).

At the same time, new cracks were visible, and there was a significant expansion of cracks after both the moisture and temperature test. This is closely related to the increased porosity of the pellets and the increase in moisture content. Pellets that have more visible cracks on their surface or within their structure tend to degrade more easily during storage. Cutz et al.^15^ observed a similar effect for woody pellets after storing them for a month at 40 °C and 85% RH. The same behavior was seen by^4^ when pellets were stored outdoors in uncovered storage during the warmer summer months.

In addition, the fresh pellets had a smooth and glossy/glassy outer surface, and after environmental testing, this degraded, revealing a rough and cracked appearance to the pellet surface.

#### Leachability of harmful components

To assess the potential impact of storage conditions on the leaching of heavy metals, the leachability test method EN 12457-4:2006, dedicated to waste characterization, was applied. This method involves stringent conditions such as soaking for 24 h and shaking during that time. These conditions can also lead to pellet abrasion and the exposure of new surfaces, from which harmful components may be leached out due to the action of water. In principle, such conditions would not be present during waste storage, so it can be assumed that the levels of heavy metal leaching obtained in this methodology will be maximum. Table 2 presents the leachability of components from the pellets, considering the variable storage conditions (humidity, variable air temperature).

**Table 2:** Leachability of components from pellets.

Parame-ter	Pellet A			Pellet B			Pellet C			Pellet D		
	Fresh	Humidity test	Temperature test	Fresh, mg/kg	Humidity test	Temperature test	Fresh	Humidity test	Temperature test	Fresh	Humidity test	Temperature test
pH	5.52 ± 0.01	5.67 ± 0.02	5.62 ± 0.02	7.37 ± 0.04	8.01 ± 0.03	7.24 ± 0.03	6.91 ± 0.03	6.87 ± 0.04	6.96 ± 0.04	4.74 ± 0.01	4.66 ± 0.02	4.82 ± 0.01
mg/kg												
As	< 0.02	0.16 ± 0.05	0.14 ± 0.03	0.22 ± 0.04	0.18 ± 0.04	0.22 ± 0. 02	0.31 ± 0.04	0.20 ± 0.04	0.25 ± 0.04	0.11 ± 0.00	0.07 ± 0.00	0.09 ± 0.02
Ba	0.43 ± 0.01	0.43 ± 0.10	0.41 ± 0.07	1.49 ± 0.40	2.14 ± 0.55	2.93 ± 0.22	1.01 ± 0.01	0.96 ± 0.10	0.71 ± 0.01	0.32 ± 0.10	0.26 ± 0.04	0.31 ± 0.11
Cd	< 0.03	< 0.03	< 0.03	< 0.03	< 0.03	< 0.03	< 0.03	< 0.03	< 0.03	< 0.03	< 0.03	< 0.03
Co	0.03 ± 0.00	< 0.03	< 0.03	< 0.03	< 0.03	< 0.03	0.09 ± 0.03	0.05 ± 0.00	0.04 ± 0.00	< 0.03	< 0.03	< 0.03
Cr	< 0.06	0.08 ± 0.03	< 0.06	0.21 ± 0.03	0.12 ± 0.03	0.13 ± 0.01	0.37 ± 0.09	0.16 ± 0.00	0.16 ± 0.04	< 0.06	< 0.06	< 0.06
Cu	0.85 ± 0.09	0.93 ± 0.06	1.14 ± 0.33	1.42 ± 0.11	0.55 ± 0.08	0.44 ± 0.15	2.22 ± 0.07	2.58 ± 0.21	2.38 ± 0.20	0.19 ± 0.04	0.09 ± 0.02	0.05 ± 0.00
Mn	0.72 ± 0.13	0.63 ± 0.05	1.22 ± 0.40	20.32 ± 4.00	14.73 ± 3.63	27.50 ± 4.01	21.45 ± 1.58	22.81 ± 1.99	20.54 ± 1.87	24.81 ± 4.08	21.68 ± 2.46	22.81 ± 4.32
Mo	0.04 ± 0.01	0.16 ± 0.02	0.04 ± 0.01	0.10 ± 0.02	0.08 ± 0.01	0.09 ± 0.03	0.24 ± 0.03	0.07 ± 0.00	0.06 ± 0.01	0.04 ± 0.01	0.03 ± 0.01	0.05 ± 0.02
Ni	0.20 ± 0.02	0.12 ± 0.04	0.19 ± 0.03	< 0.19	0.12 ± 0.01	0.62 ± 0.08	1.95 ± 0.02	1.91 ± 0.25	1.82 ± 0.22	< 0.05	< 0.05	0.99 ± 0.15
Pb	< 0.19	< 0.19	< 0.19	< 0.19	< 0.19	< 0.19	0.94 ± 0.02	< 0.19	< 0.19	< 0.19	< 0.19	< 0.19
Sb	0.08 ± 0.00	0.11 ± 0.01	0.05 ± 0.01	0.08 ± 0.00	0.09 ± 0.01	0.10 ± 0.01	0.19 ± 0.06	0.11 ± 0.00	0.09 ± 0.01	0.06 ± 0.01	0.08 ± 0.00	0.07 ± 0.01
Se	0.02 ± 0.00	0.09 ± 0.00	0.06 ± 0.01	0.04 ± 0.00	0.07 ± 0.00	0.05 ± 0.00	0.11 ± 0.03	0.09 ± 0.00	0.06 ± 0.01	0.04 ± 0.00	0.03 ± 0.00	0.03 ± 0.00
Zn	1.09 ± 0.22	0.99 ± 0.01	0.89 ± 0.17	2.86 ± 0.6	3.12 ± 0.65	3.65 ± 0.67	19.77 ± 0.41	20.11 ± 1	17.23 ± 0.20	1.69 ± 0.13	1.61 ± 0.25	1.40 ± 0.30

The main factor determining the solubility of metal compounds is the pH of the interacting environment, and the solubility of heavy metal compounds depends on it.

In alkaline and neutral pH, the process of exchange sorption (metal ions and hydrogen ions) is small, while it is much higher in acidic environments^16,17^.

The aqueous eluates from pellet leaching have a range of pH values from 4.7 to 7.0. The lowest value was obtained for Pellet D 4.66–4.82 and Pellet A 5.52–5.67. In contrast, higher values of 7.24–8.01 and 6.9–7.0 were obtained for Pellet B and C, respectively.

The effect of storage under different environmental conditions on the leachability of constituents from the pellets was not observed. The results obtained for leachability of heavy metals after environmental tests are comparable to leachability from fresh pellets. The highest values above 20 mg/kg were determined for manganese (Pellet B, Pellet C and Pellet D) and zinc above 17 mg/kg (Pellet C). The occurrence of these trace elements in leachates may be related to the high concentration of Zn, Mn and Cu in woody biomass^18^.

Demiras^19^ claimed that the levels of trace elements in biomass are influenced by plant species, growing site, age of the plant, and distance from the source of pollution. Compared to agricultural residues like wheat straw and fruit shells, wood ash generally contains higher levels of heavy metals.

It is worth noting that despite the adverse storage conditions of the pellets, a decrease in the leaching levels of certain heavy metals has been observed. These results were seen, for example, for Cr and Ni (Pellet B and Pellet C) or Cu (Pellets B and Pellet D). In general, it can be concluded that the determined leaching values of heavy metals from the pellets reach low levels and should not deteriorate the quality of soils in the vicinity of their storage, including Class I soils (which are intended not only for commercial activities but also for residential purposes)^20^.

The test on the leachability of harmful components indicates no environmental harm. However, their storage should be done under some roofing to maintain their physical properties and to avoid other potential problems.

### Mechanical properties

**Figure 3:**
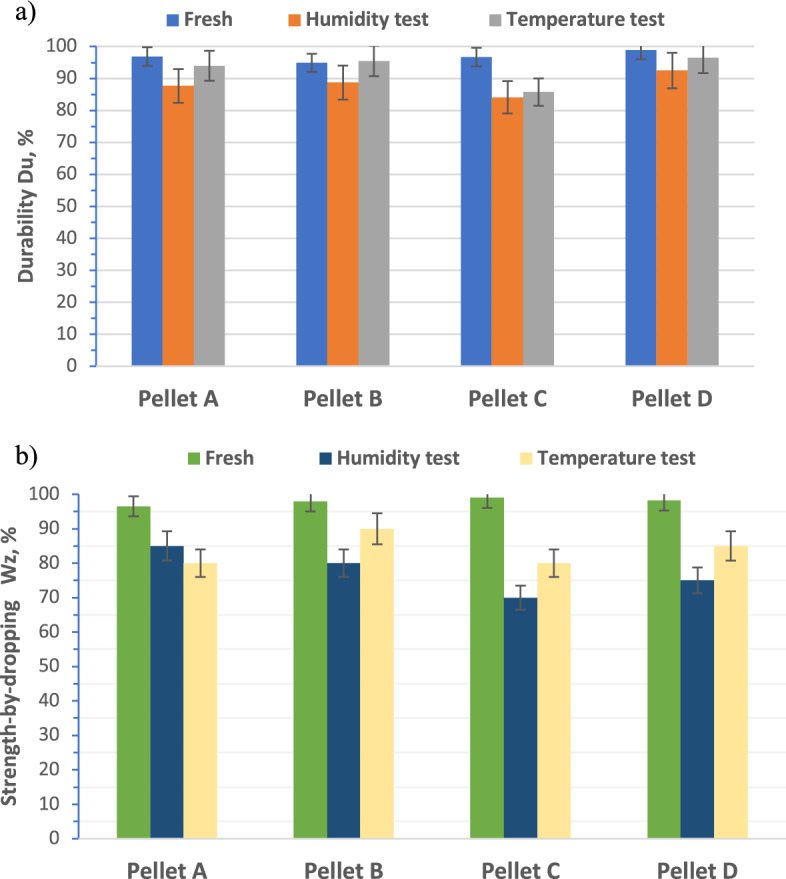
Mechanical properties: (**a**) the mechanical durability index, (**b**) strength-by-dropping index.

Mechanical durability and strength-by-dropping are two key quality parameters that indicate a pellet’s resistance to abrasion and impact. The results of these indexes for the tested pellets are shown in Fig. 3.

Tests carried out showed that pellets made from agricultural biomass have lower durability parameters than woody pellets (Pellet D), for which the durability index is 98.95% (Fig. 3a).

Nielsen et al.^21^ and González-Peña et al.^22^ suggested that higher levels of extractives present in agro-biomass compared to woody biomass caused difficulties for particles to form close contact of bonding sites compared to woody biomass, leading to low compression strength of pellets. In addition, they also have a lower lignin content than woody biomass, and according to Pradhan et al.^4^ lignin helps in building solid bridges which produces more durable pellets.

For example, hemp hurds pellet (Pellet C) had the highest durability value of pellets made from agricultural biomass, after manufacture (fresh)—96.70%, while sunflower husks pellet (Pellet A) and wheat straw pellet (Pellet B) reached 96.43% and 94.89%, respectively.

Literature^2^ reports that durability of pellets produced from cereal residues such as barley, oilseed rape oat, wheat is less than 95%, and durability of pearl millet pellets about 83% but with 50% additives (eucalyptus and corncob) it can be increased to over 95%^23^.

While the strength-by-dropping index for all types of pellets after manufacture (fresh) is within 97–98%—Fig. 3b.

The results show that all types of argo-biomass pellets tested for storage at 30 °C and 85% relative humidity (humidity test) showed significant degradation.

The pellets degraded to a less extent at varying  ± temperatures (temperature test) than at high humidity. After freezing and defreezing Pellet A and Pellet D, their durability drops by 2%, while it drops by 11% for Pellet C. The phase change of water from liquid to ice in solid materials can result in increasing their volume which causes local damages in the material such as delamination, cracks or spalling^24^.

### Energy properties

Table 3 shows the energy properties of the pellets before and after environmental testing. The pellets tested have the higher heating value (HHV) in the range of 16.7–19.2 MJ/kg, which is characteristic of plant biomass^25^.

**Table 3:** Energy properties of pellets.

Parameter	Pellet A			Pellet B			Pellet C			Pellet D		
	Fresh	H test	T test	Fresh	H test	T test	Fresh	H test	T test	Fresh	H test	T test
Moisture_a_, %	8.64 ± 0.18	8.87 ± 0.16	7.13 ± 0.16	8.06 ± 0.10	9.80 ± 0.13	7.13 ± 0.16	5.74 ± 0.15	9.16 ± 0.23	6.31 ± 0.25	7.06 ± 0.36	8.65 ± 0.13	6.69 ± 0.27
HHV_db_, kJ/kg	19,249 ± 107	18,651 ± 60	18,879 ± 54	17 811 ± 80	17,336 ± 223	16,706 ± 43	16 709 ± 98	16 515 ± 142	17 025 ± 177	18,868 ± 52	18 603 ± 133	18 867 ± 70
Ash_db_, %	3.06 ± 0.32	2.98 ± 0.37	3.00 ± 0.31	5.20 ± 0.15	4.99 ± 0.10	5.36 ± 0.08	5.80 ± 0.06	5.89 ± 0.03	5.95 ± 0.06	0.30 ± 0.06	0.29 ± 0.07	0.30 ± 0.04
Voltaire matter_db_, %	77.62 ± 0.20	77.28 ± 0.03	77.05 ± 0.08	77.62 ± 0.1	77.89 ± 0.12	76.46 ± 0.12	77.41 ± 0.12	77.69 ± 0.10	77.35 ± 0.19	84.74 ± 0.14	85.17 ± 0.19	84.86 ± 0.17
Elementary composition, % _db_												
C	52.25 ± 0.33	51.52 ± 0.32	51.44 ± 0.33	47.80 ± 0.32	47.12 ± 0.34	46.94 ± 0.33	46.16 ± 0.33	46.44 ± 0.34	46.61 ± 0.32	51.07 ± 0.33	51.26 ± 0.31	51.01 ± 0.33
H	6.95 ± 0.20	6.95 ± 0.18	6.56 ± 0.08	7.15 ± 0.19	7.10 ± 0.21	6.71 ± 0.21	6.56 ± 0.18	7.00 ± 0.21	6.54 ± 0.10	6.94 ± 0.72	7.23 ± 0.10	7.42 ± 0.72
N	0.87 ± 0.06	0.71 ± 0.06	0.70 ± 0.07	0.82 ± 0.12	0.73 ± 0.12	0.77 ± 0.12	1.24 ± 0,07	1.11 ± 0.07	1.10 ± 0.06	0.13 ± 0.06	0.10 ± 0.06	0.12 ± 0.06
S	0.10 ± 0.03	0.12 ± 0.02	0.12 ± 0.03	0.10 ± 0.02	0.09 ± 0.02	0.11 ± 0.02	0.15 ± 0.03	0.09 ± 0.02	0.07 ± 0.02	0.05 ± 0.03	0.02 ± 0.03	0.03 ± 0.02
O	39.82 ± 0.41	40.70 ± 0.41	41.18 ± 0.36	44.13 ± 0.41	44.96 ± 0.42	45.47 ± 0.41	45.89 ± 0.43	45.36 ± 0.43	45.68 ± 0.43	41.81 ± 0.43	41.39 ± 0.39	41.42 ± 0.38

H test - humidity test; T test - temperature test, HHV - Higher Heating Value; a analytical state; db - dry basis; wb - wet basis.

After the environmental tests, it can be concluded that the energy parameters, in dry mass, have not changed. Assuming the increase in moisture absorbed by the pellets during testing, and which is factored into the lower heating value (LHV), it can be concluded that their energy potential has decreased.

The calorific value linearly decreases as the moisture content of the fuel increases, as some of the heat generated is lost to heating and evaporation of moisture. In addition, wet fuel causes the flame cone to shift towards the upper part of the combustion chamber, affecting the heat transfer both in the combustion chamber and the convective section of the boiler, thereby reducing the energy efficiency of the process.

### SEM images of pellets

**Figure 4:**
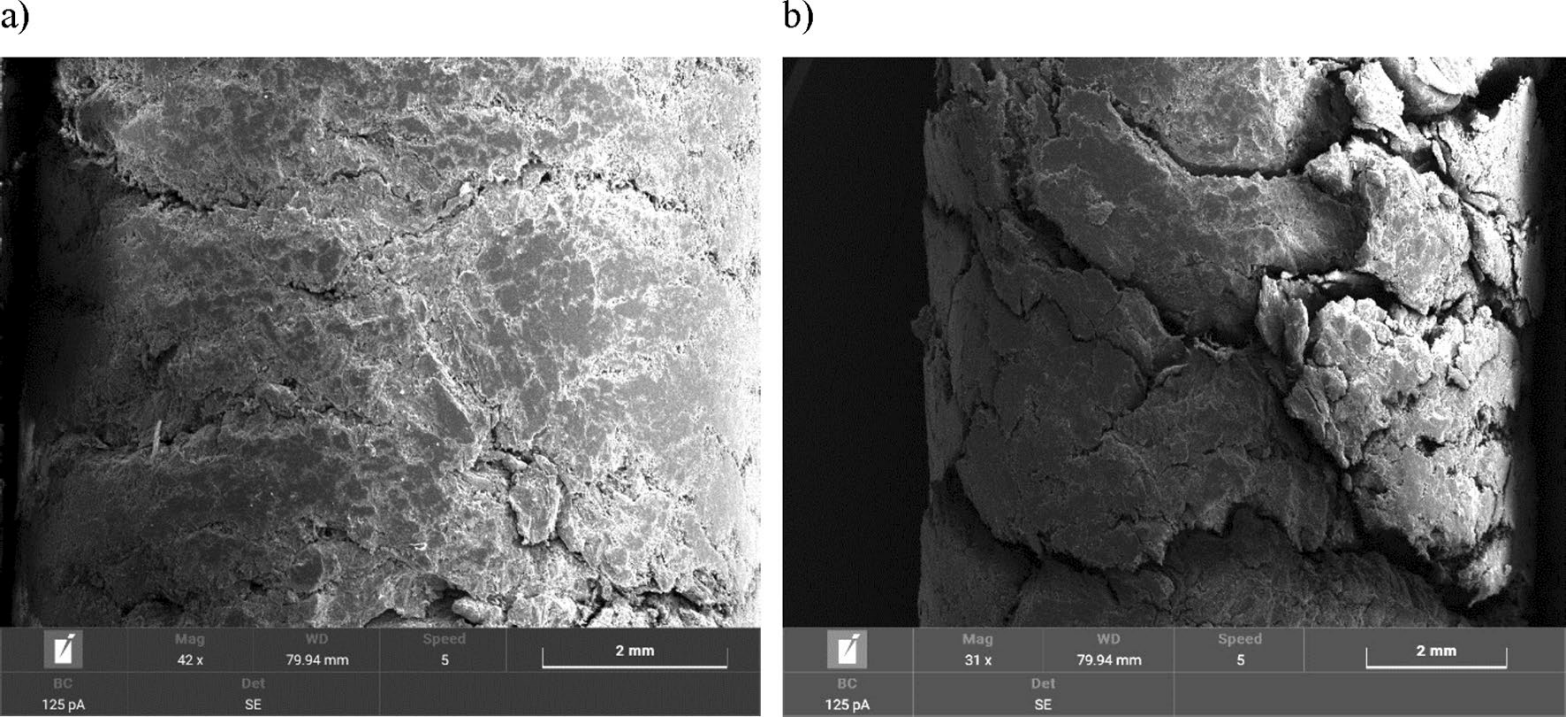
Pellet A: (**a**) surface area of fresh pellets; (**b**) surface area of pellets after moisture test.

**Figure 5:**
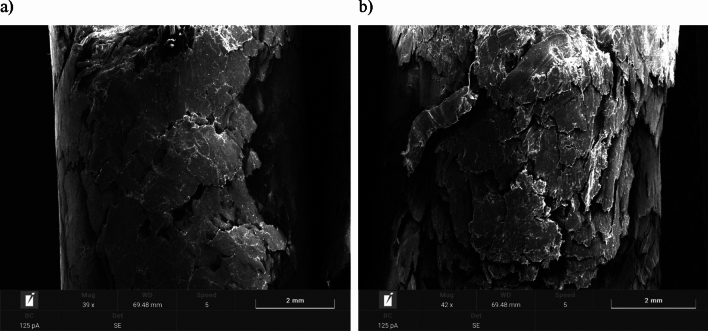
Pellet B: (**a**) surface area of fresh pellets; (**b**) surface area of pellets after moisture test.

**Figure 6:**
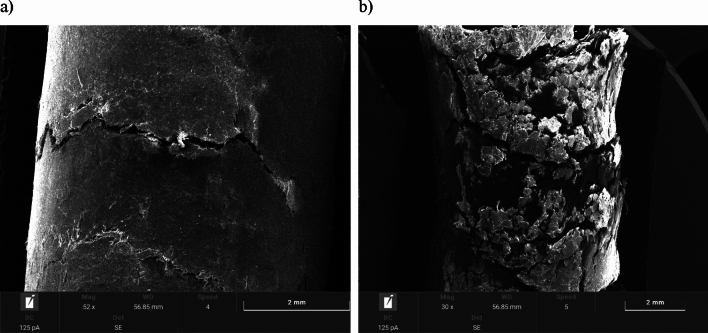
Pellet C: (**a**) surface area of fresh pellets; (**b**) surface area of pellets after moisture test.

**Figure 7:**
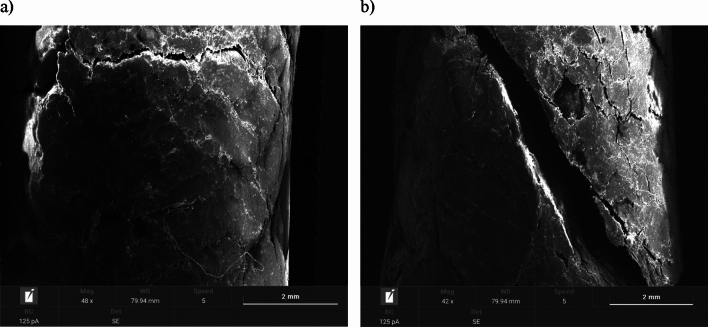
Pellet D: (**a**) surface area of fresh pellets; (**b**) surface area of pellets after moisture test.

SEM images of the surfaces of the studied pellets are shown in Figs. 4, 5, 6 and 7. All observed pellet surfaces are characterized by an irregular structure with high inhomogeneity, irregularities and depressions.

The surface morphology of fresh pellets differs noticeably from pellets after the environmental test. The surfaces of the fresh pellets have a compact structure, while the pellets after the moisture test are characterized by the presence of numerous open channels that may have formed due to moisture absorption. Excessive moisture content weakens hydrogen bonds and van der Waals forces, due to an increase in the distance between particles, resulting in the appearance of cracks and delamination, as well as the expansion of already existing cracks.

Similar phenomena were noted for both agro-biomass pellets (Pellet A, Pellet B and Pellet C) and woody biomass Pellet D.

## Conclusions

Due to the high demand for biomass as a renewable energy source, attention should be paid not only to the use of woody biomass, but also to agricultural biomass, which represents enormous energy potential.

Converting various types of agro-biomass into pellets allows an increase in their energy density and makes them easier to transport. However, it is important to take into account the fact that pellets may be subject to variable conditions during storage, which may lead to their physical and chemical degradation.

The results show that after 2 days of seasoning at 30 °C and 90%RH, all pellets became saturated with moisture. In addition, the amount of absorbed moisture was found to depend on the type of pellets. Agro-biomass pellets have a higher water absorption capacity than wood pellets.

Exposure of agro-pellets to changing weather conditions causes them to swell and delaminate their structure and reduces their mechanical strength. The pellets degraded less at varying ± temperatures than at high humidity. After freezing and defreezing, for sunflower husk pellets and wood biomass, the durability drops by 2%, while for hemp hurd pellets it drops by 11%. On the other hand, when fully saturated with moisture, the durability drops for agro-pellets by an average of 9%. The highest degradation of durability parameters was observed for hemp hurds pellets.

No change in energy parameters, i.e. HHV or elemental composition of pellets in dry matter, was noticed. On the other hand, an increase in the moisture content of pellets during storage under different environmental conditions results in a decrease in heating value, which can have significant implications for the thermal efficiency of the combustion process.

The results of the study showed higher degradability of agro-biomass pellets compared to woody biomass, so it is important to secure appropriate storage conditions for agro-biomass pellets.

## Data Availability

All data generated or analysed during this study are included in this published article.
